# Supplementary material for: Resolving the full spectrum of human genome variation using Linked-Reads
Source: Genome Res. 2019 Apr;29(4):635–45. doi: 10.1101/gr.234443.118 (PMC6442396; doi:10.1101/gr.234443.118)
Supplement: Supplemental Material [file supp_gr.234443.118_Supplemental_longranger-2.2.zip › longranger-2.2.tar.bz2!/lr2.2-public/lib/stan/cmdstan/stan/doc/api/html/tail_8hpp_source.html]

Stan: src/stan/math/matrix/tail.hpp Source File


|  |  |
| --- | --- |
|  | Stan  2.5.0  probability, sampling & optimization |


- Main Page
- Related Pages
- Namespaces
- Classes
- Files

- File List
- File Members

All Classes Namespaces Files Functions Variables Typedefs Enumerations Enumerator Friends Macros Pages

- src
- stan
- math
- matrix

tail.hpp

Go to the documentation of this file.

1 #ifndef STAN\_\_MATH\_\_MATRIX\_\_TAIL\_HPP

2 #define STAN\_\_MATH\_\_MATRIX\_\_TAIL\_HPP

3

4 #include <vector>

5 #include <stan/math/error\_handling/matrix/check\_column\_index.hpp>

6 #include <stan/math/error\_handling/matrix/check\_row\_index.hpp>

7 #include <stan/math/error\_handling/matrix/check\_std\_vector\_index.hpp>

8 #include <stan/math/matrix/Eigen.hpp>

9 #include <stan/math/matrix/meta/index\_type.hpp>

10

11 namespace stan {

12

13  namespace math {

14

19  template <typename T>

20  inline

21  Eigen::Matrix<T,Eigen::Dynamic,1>

22  tail(const Eigen::Matrix<T,Eigen::Dynamic,1>& v,

23  size\_t n) {

24  if (n != 0)

25  stan::math::check\_row\_index("tail(%1%)",n,v,"n",(double\*)0);

26  return v.tail(n);

27  }

28

29

34  template <typename T>

35  inline

36  Eigen::Matrix<T,1,Eigen::Dynamic>

37  tail(const Eigen::Matrix<T,1,Eigen::Dynamic>& rv,

38  size\_t n) {

39  if (n != 0)

40  stan::math::check\_column\_index("tail(%1%)",n,rv,"n",(double\*)0);

41  return rv.tail(n);

42  }

43

44  template <typename T>

45  std::vector<T> tail(const std::vector<T>& sv,

46  size\_t n) {

47  typedef typename index\_type<std::vector<T> >::type idx\_t;

48  if (n != 0)

49  stan::math::check\_std\_vector\_index("tail(%1%)",n,sv,"n",(double\*)0);

50  std::vector<T> s;

51  for (idx\_t i = sv.size() - n; i < sv.size(); ++i)

52  s.push\_back(sv[i]);

53  return s;

54  }

55

56

57  }

58 }

59

60 #endif

stan::math::check\_std\_vector\_index

bool check\_std\_vector\_index(const char \*function, size\_t i, const std::vector< T\_y > &y, const char \*name, T\_result \*result)

Return true if the specified index is valid in std vector.

**Definition:** check\_std\_vector\_index.hpp:25

check\_column\_index.hpp

stan::math::check\_column\_index

bool check\_column\_index(const char \*function, size\_t i, const Eigen::Matrix< T\_y, R, C > &y, const char \*name, T\_result \*result)

Return true if the specified index is a valid column of the matrix.

**Definition:** check\_column\_index.hpp:25

check\_std\_vector\_index.hpp

check\_row\_index.hpp

stan::math::index\_type

Primary template class for the metaprogram to compute the index type of a container.

**Definition:** index\_type.hpp:21

stan::math::tail

Eigen::Matrix< T, Eigen::Dynamic, 1 > tail(const Eigen::Matrix< T, Eigen::Dynamic, 1 > &v, size\_t n)

Return the specified number of elements as a vector from the back of the specified vector...

**Definition:** tail.hpp:22

index\_type.hpp

stan::math::check\_row\_index

bool check\_row\_index(const char \*function, size\_t i, const Eigen::Matrix< T\_y, R, C > &y, const char \*name, T\_result \*result)

Return true if the specified index is a valid row of the matrix.

**Definition:** check\_row\_index.hpp:25

Eigen.hpp


---

[ Stan Home Page ]

*© 2011–2014,
Stan Development Team.*
